# A prominent role of LncRNA H19 in* H. pylori* CagA induced DNA damage response and cell malignancy

**DOI:** 10.1038/s41598-024-65221-y

**Published:** 2024-06-20

**Authors:** Xiaofeng He, Tingting Huang, Qinrong Wang, Liya Bao, Zhengrong Wang, Hui Song, Yanhong Li, Jianjiang Zhou, Yan Zhao, Yuan Xie

**Affiliations:** 1 Key Laboratory of Endemic and Ethnic Minority Diseases, Ministry of Education and Key Laboratory of Molecular Biology, Guizhou Medical University, 4 Beijing Road, Guiyang, 550004 Guizhou People’s Republic of China; 2 Affiliated Hospital of Zunyi Medical University, 149 Dalian Road, Huichuan District, Zunyi, 563003 Guizhou People’s Republic of China; 3 Hepatitis Laboratory, Department of Infectious Diseases, Affiliated Hospital of Guizhou Medical University, Guiyang, 550004 Guizhou People’s Republic of China; 4 School of Clinical Laboratory Science, Guizhou Medical University, Guiyang, 550004 Guizhou People’s Republic of China

**Keywords:** DNA damage response, Gastric cancer, *Helicobacter pylori* (*H. pylori*), LncRNA H19, Migration, Invasion, Cancer, Cell biology, Microbiology, Molecular biology, Gastroenterology, Molecular medicine

## Abstract

*Helicobacter pylori* (*H. pylori*), together with its CagA, has been implicated in causing DNA damage, cell cycle arrest, *ap*optosis, and the development of gastric cancer. Although lncRNA H19 is abundantly expressed in gastric cancer and functions as a pro-oncogene, it remains unclear whether lncRNA H19 contributes to the oncogenic process of *H. pylori* CagA. This study investigates the role of H19 in the DNA damage response and malignancy induced by *H. pylori*. It was observed that cells infected with CagA^+^ *H. pylori* strain (GZ7/cagA) showed significantly higher H19 expression, resulting in increased *γH2A.X* and p-ATM expression and decreased p53 and Rad51 expression. Faster cell migration and invasion was also observed, which was reversed by H19 knockdown in *H. pylori*. YWHAZ was identified as an H19 target protein, and its expression was increased in H19 knockdown cells. GZ7/cagA infection responded to the increased YWHAZ expression induced by H19 knockdown. In addition, H19 knockdown stimulated cells to enter the G2-phase and attenuated the effect of GZ7/cagA infection on the cellular S-phase barrier. The results suggest that *H. pylori* CagA can upregulate H19 expression, participate in the DNA damage response and promote cell migration and invasion, and possibly affect cell cycle arrest via regulation of YWHAZ.

## Introduction

*H. pylori* infection is the leading cause of gastric cancer (GC), accounting for more than 60% of all cases^1^. *H. pylori* employs several virulence factors to affect host proliferation, apoptosis, migration, and inflammatory responses, resulting in a complex biological process that leads to GC^2^. CagA is a well-established oncoprotein secreted by the *H. pylori* type IV secretion system (T4SS) into the gastric epithelial cells. It either stimulates or inhibits the intracellular signalling pathways and thus plays a role in GC formation. However, the specific mechanism by which it causes cancer is not yet understood^3^.

Long non-coding RNAs (lncRNAs) are RNA molecules that are longer than 200 nucleotides (nt) and lack a well-defined open reading frame. They have been found to control various physiological processes, such as inflammatory responses, antiviral defence mechanisms, and DNA damage repair^4^. Therefore, it is essential to understand the role of lncRNAs in cancer, especially their impact on epigenetic regulation, DNA damage, cell cycle regulation, microRNA regulation, signal transduction, and mediation of hormone-induced cancer^5^. LncRNA H19 (H19), the first LncRNA identified, is highly expressed in many types of cancers and plays an essential role in the regulation of several biological processes, including tumour cell proliferation, metastasis, metabolism, and autophagy^6^. H19 functions through a variety of mechanisms, including interactions with miRNAs and/or target proteins to maintain cancer characteristics. H19 can also compete with mRNAs by antagonising miRNAs, revealing a regulatory network model of “H19-miRNAs-mRNAs”^7^. For example, overexpression of H19 was found to promote cell growth by regulating p53 in GC^8^. Li et al.^9^ showed that the effects of H19 were partly through direct upregulation of ISM1 and indirect downregulation of CALN1 via miR-675. Similar results indicated that H19 could modulate GC progression through the miR-675/RUNX1 pathway, revealing a potential target for GC therapy^10^. A study showed that H19 expression was higher in the serum of *H. pylori*-infected GC patients^11^. Furthermore, overexpression of H19 in *H. pylori*-infected GC cells promoted inflammation by increasing nuclear factor-kappa B (NF-κB) and enhancing cell proliferation and invasion^12^. Therefore, *H. pylori* may be involved in the development of GC by increasing H19 expression.

Various endogenous and exogenous factors can cause DNA damage, and DNA damage must be repaired by activating DNA damage repair pathways to maintain genomic stability^13^. Defective or imbalanced DNA damage repair function is a major cause of cell cycle arrest, aberrant autophagy, and cell death, leading to abnormal cell proliferation, increased ability to migrate, invasion, and tumour cell formation^14^. DNA double-strand breaks (DSBs) represent the most severe type of DNA damage, and their proper repair is primarily dependent on DNA damage response (DDR) and homologous recombination (HR) repair processes. Several key proteins, including γH2A.X, p53, ATM, and Rad51, play critical roles in the DSB repair pathway^15,16^. *H. pylori* has been shown to induce DSBs in gastric epithelial cells in order to modulate the DDR mechanism^17^. For example, a study by Kontizas et al.^18^ reported that persistently activated inflammation caused by CagA^+^ *H. pylori* infection can significantly increase reactive oxygen and nitrogen species (RONS) levels, resulting in oxidative DNA damage. Recent studies have shown that lncRNAs, as regulators of DDR, play an essential role in initiating the DDR process^19–21^. In addition, lncRNAs can efficiently control the response to DNA damage and repair by modifying the ATM/ATR, the p53 regulatory network, and the DNA double-strand break repair pathway^22^. Currently, only a few studies have investigated the role of H19 in DDR, and the underlying processes remain unclear.

Therefore, this study investigated the effects of *H. pylori* and its CagA on H19 expression and the role of H19 in DDR, GC cell proliferation, and invasive potential of *H. pylori* CagA-induced cells. The results showed that *H. pylori* CagA can increase the expression of H19, participate in DNA damage response, and promote proliferation and invasion of GC cells. YWHAZ may function as an H19 binding protein and contribute to the cell cycle arrest caused by *H. pylori* CagA. These findings provide new insights into the molecular mechanism of *H. pylori* CagA-induced GC.

## Materials and methods

### *H. pylori *strains, cell lines, and infection methods

The human gastric epithelial adenocarcinoma cell lines AGS and SGC7901, GZ7/cagA (we identified CagA^+^*H. pylori* wild strain, GenBank accession ID: KR154737.1) and GZ7/ΔcagA (cagA gene knockout from the GZ7/cagA was constructed and identified by Sangon Biotech, Shanghai, China) were cultured and infected according to the methods described in previous research work^21^.

### RNA FISH

RNA fluorescence in situ hybridisation (FISH) technique was used to detect the subcellular localisation of H19. AGS and SGC-7901 cells were grown on a glass slide and then infected with GZ7/ΔcagA or GZ7/cagA at the multiplicity of infection (MOI) of 60. The experiment was performed according to the instructions of the FISH kit (GenePharma Inc, China). Once the cells reached 80% confluence, they were rinsed with PBS and then treated with 4% formaldehyde at room temperature for fixation. The cells were then washed again with PBS, permeabilized with Triton X-100, dehydrated through a series of ethanol washes and hybridised overnight with a digoxigenin-labelled H19 probe. Confocal microscopy (Carl Zeiss LSM 880, Germany) was utilized to examine the RNA FISH signal, which was detected by incubation with an Alexa Fluor 647-labelled anti-digoxigenin antibody.

### RT-qPCR assay

Cells from the study groups were harvested and total RNA was extracted using TRIzol reagent (Invitrogen, USA). The Transcriptor First Strand cDNA Synthesis Kit (Roche, USA) was used to reverse transcribe the RNA into cDNA. FastStart Universal SYBR Green I Master (ROX, Roche, USA) and CFX Connect PCR System (Bio-Rad, USA) were used for qRT-PCR according to the manufacturer’s instructions. GAPDH was used as internal reference. The expression of H19 and YWHAZ was determined by the 2−∆∆Ct method and normalised to GAPDH. The sequence of all primers (Sangon, Biotech, China) used in this study is shown in Table 1.

**Table 1:** The primers for real-time reverse transcription-polymerase chain reaction.

Gene	Primer sequences (5′–3′)
H19	F-GAAGGCCAAGACGCCAGG R-TCCTCTGTCCTCGCCGTCAC
YWHAZ	F-ATTGAACAAAAGACGGAA
	R-CAGCCAAGTAACGGTAGTA
GAPDH	F-AGAAGGCTGGGGCTCATTTG R-AGGGGCCATCCACAGTCTTC

### PcDNA3.1-H19 plasmid, si-H19, and cell transfection

The pcDNA3.1-H19, empty pcDNA3.1, H19 targeting siRNA (si-H19), and H19 negative control (si-ctrl) were obtained from GenePharma Inc. (China). The lncRNA H19 siRNA sequences were as follows: si-H19, 5′-CCCGUCCCUUCUGAAUUUATT-3′ and si-ctrl, 5′-UUCUUCGAA CGUGUCACGUTT-3′. Lipofectamine 2000 (Invitrogen) was utilized for transfection according to the manufacturer’s instructions. The transfected cells were collected 24 h later and used in the subsequent experiments.

### γH2A.X Immunofluorescence assay

AGS and SGC-7901 cells were cultured on a glass slide and treated with pcDNA3.1-H19, si-H19, and *H. pylori*, respectively. The cells on the slides were permeabilized with 0.5% Triton X-100 for 50 min at room temperature, followed by fixation with 4% paraformaldehyde for 30 min at 4 ℃. Cells were then blocked with 1% bovine serum albumin (BSA) for 1 h before being treated with γH2A.X primary antibodies (1:200; ab188819, Abcam, UK) overnight at 4 ℃. The following day, cells were incubated with fluorescein isothiocyanate (FITC)-conjugated secondary antibodies (1:300; SA00003-2, Proteintech, USA) for 2 h at room temperature before washing with PBS. DAPI was used for nuclear counterstaining. Stained slides were imaged and analysed using a Carl Zeiss LSM 880 confocal microscope and Image J software (version 1.41; National Institutes of Health), respectively.

### Real-time impedance-based cell analysis

The instrument chamber (E‐plate16, ACEA Biosciences) was seeded with AGS and SGC-7901 cells (5 × 10^3^ cells/well) and allowed to grow for 24 h before treatment with si-H19 transfection and *H. pylori* infection. Proliferation and size fluctuations were monitored in real-time and recorded as impedance using an xCELLigence Real‐Time Cell Analysis System (RTCA; ACEA Biosciences, CA, USA). Subsequently, Cells were then monitored for 72 h, during which time proliferation was assessed by measuring electrical impedance using gold microelectrodes. The impedance was used to calculate the cell index value related to cell growth, which was measured every 10 min for 72 h using the RTCA program (ACEA Biosciences).

### Wound healing assay

AGS and SGC-7901 cells (1 × 10^6^ cells/well) were grown overnight on a 6-well plate. At approximately 90% confluence, a scratch was made in the centre of the well using a 200 µL pipette tip. The cells were then treated with pcDNA3.1-H19, si-H19, and *H. pylori*. The wounded cells were then subsequently rinsed with PBS and cultured for 48 h in a medium containing 4% fetal bovine serum (FBS). Finally, wound closure was observed as previously described^23^.

### Cell migration and invasion assay

Cell migration and invasion assays were performed in a 24-well Transwell plate with 8 μm pore size (Costar; Corning, Inc.). The cells were treated with pcDNA3.1-H19, si-H19, and *H. pylori*. After 48 h of treatment, the migratory and invasive abilities of the cells were assessed using the previously reported method^23^.

### RNA pull-down and mass spectrometry identification

Sense and antisense H19 strands were obtained from the pcDNA3.1-H19 plasmid by PCR technique. Biotin-labelled RNAs of H19 were synthesised in vitro using the Biotin RNA Labelling Mix and T7 RNA polymerase (cat. no. 20163; Thermo Fisher Scientific, Inc.USA). The RNA pull-down experiment was performed using the PierceTM Magnetic RNA–Protein Pull-Down kit (Cat. No. 20164; Thermo Fisher Scientific, Inc. USA) according to the manufacturer’s instructions. Mass spectrometry (Sangon, Biotech, China) was used to determine the protein-binding complexes of lncRNA from the RNA pull-down experiment.

### Cell cycle analysis

AGS cells were first transfected with si-H19 and then infected with H. pylori. The cells were then fixed in 75% ice cold ethanol overnight. After two washes with PBS, the propidium iodide (PI)-treated cells were thoroughly rinsed with PBS according to the manufacturer’s instructions of the cell cycle analysis kit (KGA512, Keygen, China). The cell cycle was detected by flow cytometry (FACS verse, BD Bioscience, USA).

### Western blot analysis

Western blot analysis was performed as described in the previous publication^24^. The following antibodies were used: anti-CagA (1:500; sc-28368, Santa Cruz, USA), anti-Urease B (1:30,000; ab127916, Abcam, UK), anti-γH2A.X (1:200; ab188819, Abcam, UK), anti-pATM (1:20,000; ab81292, Abcam, UK), anti-p53 (1:1000; ab131442, Abcam, UK), anti-Rad51 (1:2000; ab63801, Abcam, UK), anti-YWHAZ antibody (1:4000; 14881-1-AP, Proteintech, USA), anti-β-actin (1:20,000; 10494-1-AP, Proteintech, USA), and HRP-conjugated Goat Anti-Rabbit secondary antibody (1:5000; SA00001-2, Proteintech, USA). The images of the protein bands were captured using a chemiluminescence imager (Amersham Biosciences, USA) along with an ECL reagent (Thermo Fisher Scientific, MA, USA). Finally, the intensities of the Western blot bands were analysed using ImageJ software, with β-actin protein acting as a loading control.

### Statistical analysis

Experiments were performed three times, and the data analysis was performed using Statistical Package for the Social Sciences (SPSS) software version 16.0 (IBM, Armonk, NY, USA), ImageJ software version 1.41 (National Institutes of Health), and GraphPad Prism software version 8.0.2 (La Jolla, CA, USA). Data were expressed as mean ± standard deviation (SD). We examined the differential expression and Kaplan–Meier (K–M) overall survival (OS) of H19 in the GEPIA II database (http://gepia2.cancer-pku.cn/). The survival results were presented as hazard ratios (HRs) and *p* values from a log-rank test. The* p* value cut-off was 0.01. The correlation of H19 expression with clinicopathological features of gastric cancer was further investigated using The Cancer Genome Atlas (TCGA) dataset (https://xenabrowser.net/datapages/), using the χ^2^ test. Student’s t-test or one-way analysis of variance (ANOVA) was used to determine statistical significance. The level of statistical significance was set at *p* < 0.05.

## Results

### H19 expression and cytoplasmic localisation were increased in *H. pylori* infected cells

**Figure 1:**
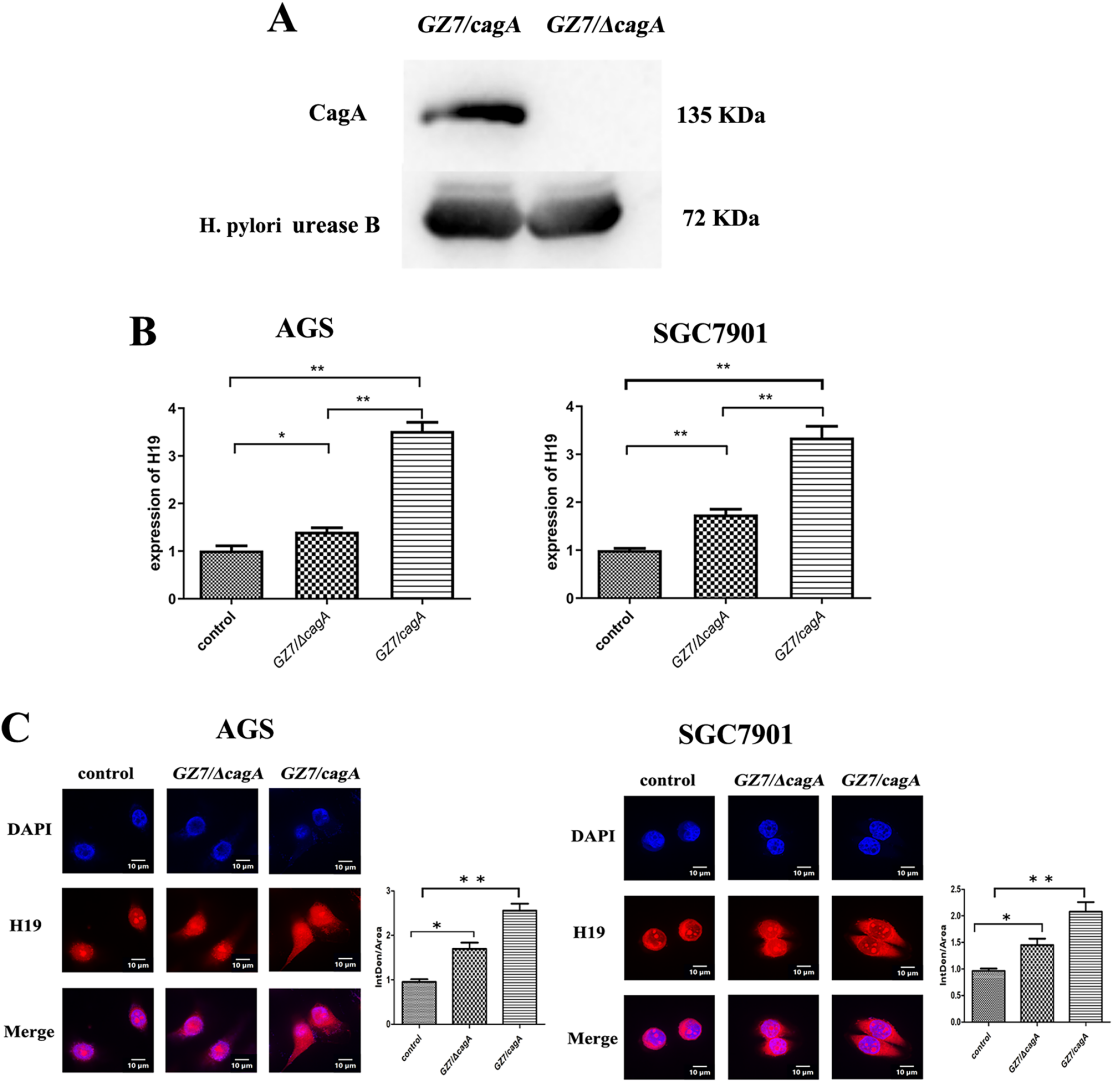
Expression and localization of H19 after GZ7/cagA and GZ7/ΔcagA infection. AGS and SGC7901 cells were infected with GZ7/ΔcagA and GZ7/cagA strains for 24 h at an MOI of 60. (**A**) Western Blot results indicated that CagA protein expression was absent in the GZ7/ΔcagA strain; (**B**) qRT-PCR was used to investigate H19 expression in cells infected with GZ7/cagA strains and GZ7/ΔcagA strains; (**C**) FISH was employed to locate H19 in cells infected with GZ7/cagA and GZ7/ΔcagA. Each experiment was performed in triplicate. *Control* non-infected cells, *GZ7/ΔcagA* the GZ7/ΔcagA strains infected cells, *GZ7/cagA* the GZ7/cagA strains infected cells; *p < 0.05, **p < 0.01; GZ7/cagA vs control, GZ7/cagA vs GZ7/ΔcagA.

To investigate the effect of *H. pylori* infection on H19 expression and the potential role of CagA in this process, the AGS and SGC7901 cells were infected with GZ7/ΔcagA and GZ7/cagA strains (MOI 60:1) for 24 h. Total RNA and protein were extracted from the infected cells. First, the absence of CagA protein expression in the GZ7/ΔcagA strain was verified by Western blot analysis (Fig. 1A). Subsequently, qRT-PCR analysis showed an increase in H19 expression in AGS and SGC7901 cells infected with GZ7/ΔcagA and GZ7/cagA strains. Interestingly, the upregulation of H19 was more pronounced in the GZ7/cagA infection group compared to the GZ7/ΔcagA infection group (Fig. 1B). Furthermore, FISH analysis revealed a significant increase in H19 localisation in the cytoplasm after infection with GZ7/ΔcagA and GZ7/cagA strains compared to the control group with H19 being more abundant in the cytoplasm of GZ7/cagA infected cells (Fig. 1C). Thus, CagA was found to enhance H19 expression and potentially increase its cytoplasmic localisation (Supplementary Information).

### H19 participates in the regulation of the DNA damage response

**Figure 2:**
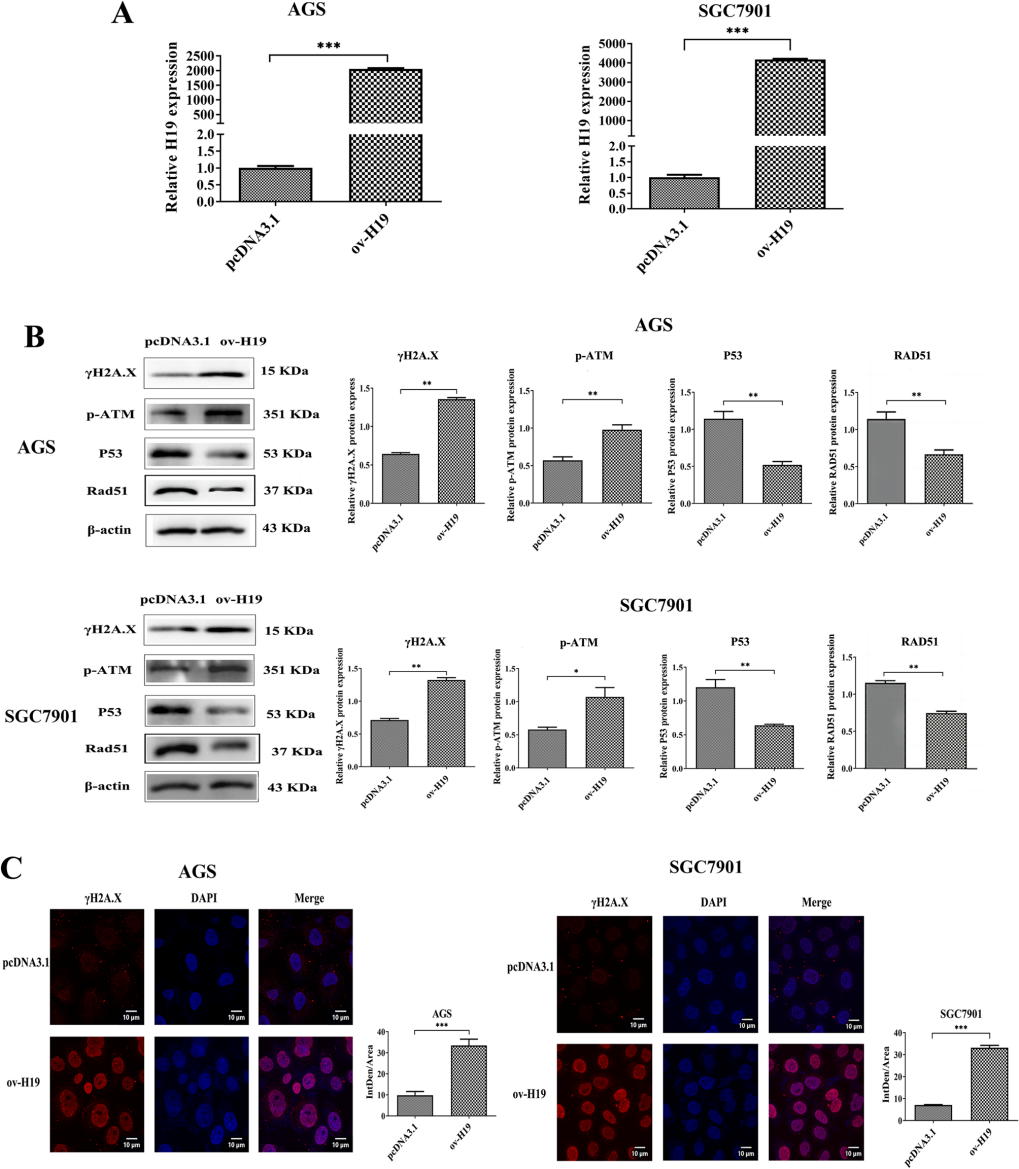
Effect of H19 overexpression on DNA damage response. AGS and SGC7901 cells were transfected with pcDNA3.1-H19 for 24 h, and total RNA and protein were extracted. (**A**) qRT-PCR was performed to verify H19 expression. (**B**) The expression of important DNA damage repair proteins γH2A.X, p-ATM, p53, and Rad51 were detected by Western Blot. (**C**) Average fluorescence intensity of γH2A.X was detected by immunofluorescence. Each experiment was performed in triplicate. *pcDNA3.1* pcDNA3.1 empty vector-transfected cells, *ov-H19* pcDNA3.1-H19 transfected cells. *p < 0.05; **p < 0.01; ***p < 0.001.

Evidence has indicated that *H. pylori* can disrupt DNA repair pathways and other responses to DNA damage^25^. In addition, previous research has shown that *H. pylori* can induce H19 overexpression. Therefore, it is necessary to investigate the potential involvement of H19 overexpression in the DNA damage response. After transfection of the pcDNA3.1-H19 into the AGS and SGC7901 cells, qRT-PCR findings results indicated a significant increase in H19 expression in the pcDNA3.1-H19 transfected cells (ov-H19) compared to the pcDNA3.1 empty vector transfected cells (pcDNA3.1; Fig. 2A). Western blot analysis was performed to investigate the effects of increased H19 expression. The results showed an up-regulation of γH2A.X and p-ATM proteins, which are crucial in the context of DNA damage. In contrast, the expression of p53 and Rad51, two proteins involved in DNA repair, was observed to be less frequent (Fig. 2B). Immunofluorescence detection of γH2A.X protein in the ov-H19 group revealed a significant increase in its average fluorescence level (Fig. 2C). These results indicated that overexpression of H19 plays an important role in the regulation of the DNA damage repair response.

### H19 can participate in the DNA damage response generated by *H. pylori* CagA

**Figure 3:**
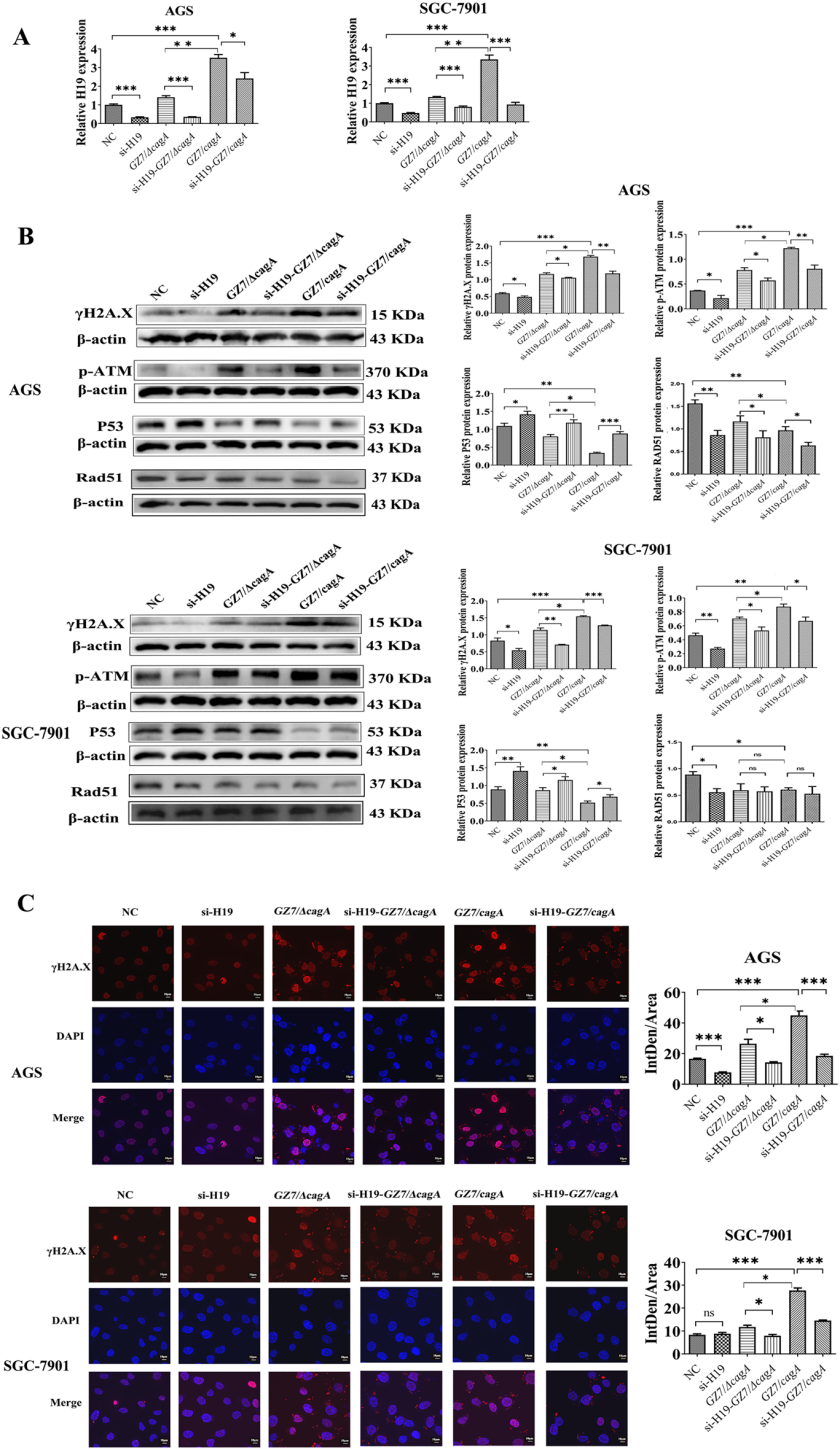
H19 knockdown could attenuate DNA damage caused by *H. pylori* CagA. After transfecting AGS and SGC7901 cells with si-H19 for 24 h, GZ7/ΔcagA and GZ7/cagA strains were infected for 24 h at an MOI of 60. (**A**) RT-qPCR for H19 expression. (**B**) Western Blot analysis for γH2A.X, p-ATM, p53, and Rad51 expression. (**C**) Immunofluorescence staining for γH2A.X expression and localization. Each experiment was conducted in triplicate. *NC* si-H19 control transfected cells, *si-H19* si-H19 transfected cells, *GZ7/ΔcagA* GZ7/ΔcagA strains infected cells, *GZ7/cagA* GZ7/cagA strains infected cells, *si-H19-GZ7/ΔcagA* GZ7/ΔcagA strains infect si-H19-transfected cells, *si-H19-GZ7/cagA* GZ7/cagA strains infect si-H19-transfected cells. *p < 0.05; **p < 0.01; ***p < 0.001.

To gain insight into the role of H19 in the DNA damage response induced by *H. pylori* CagA, AGS and SGC-7901 cells were transfected with si-H19 and subsequently infected with GZ7/cagA and GZ7/ΔcagA strains. The results of qRT-PCR indicated that H19 knockdown could significantly reduce the increased H19 expression caused by *H. pylori* infection (Fig. 3A). Western blot analysis was performed to determine the levels of γH2A.X, p-ATM, p53, and Rad51 proteins in the cells from each group. Knockdown of H19 was found to reverse the upregulated expression of γH2A.X, p-ATM, and decreased expression of p53 protein caused by *H. pylori* infections. However, the expression of Rad51 protein significantly reduced by si-H19 transfection and *H. pylori* infection, thus suggesting that H19 knockdown may minimise the DNA damage response caused by *H. pylori* infection but has minimal effect on Rad51-related DNA repair (Fig. 3B). The average fluorescence intensity of γH2A.X was assessed using the immunofluorescence assay to confirm that H19 knockdown could potentially reduce DNA damage caused by *H. pylori* infection (Fig. 3C). Overall, H19 appears to play a role in the DNA damage response mediated by *H. pylori* and CagA.

### H19 is upregulated in gastric cancer and associated with poor prognosis

**Figure 4:**
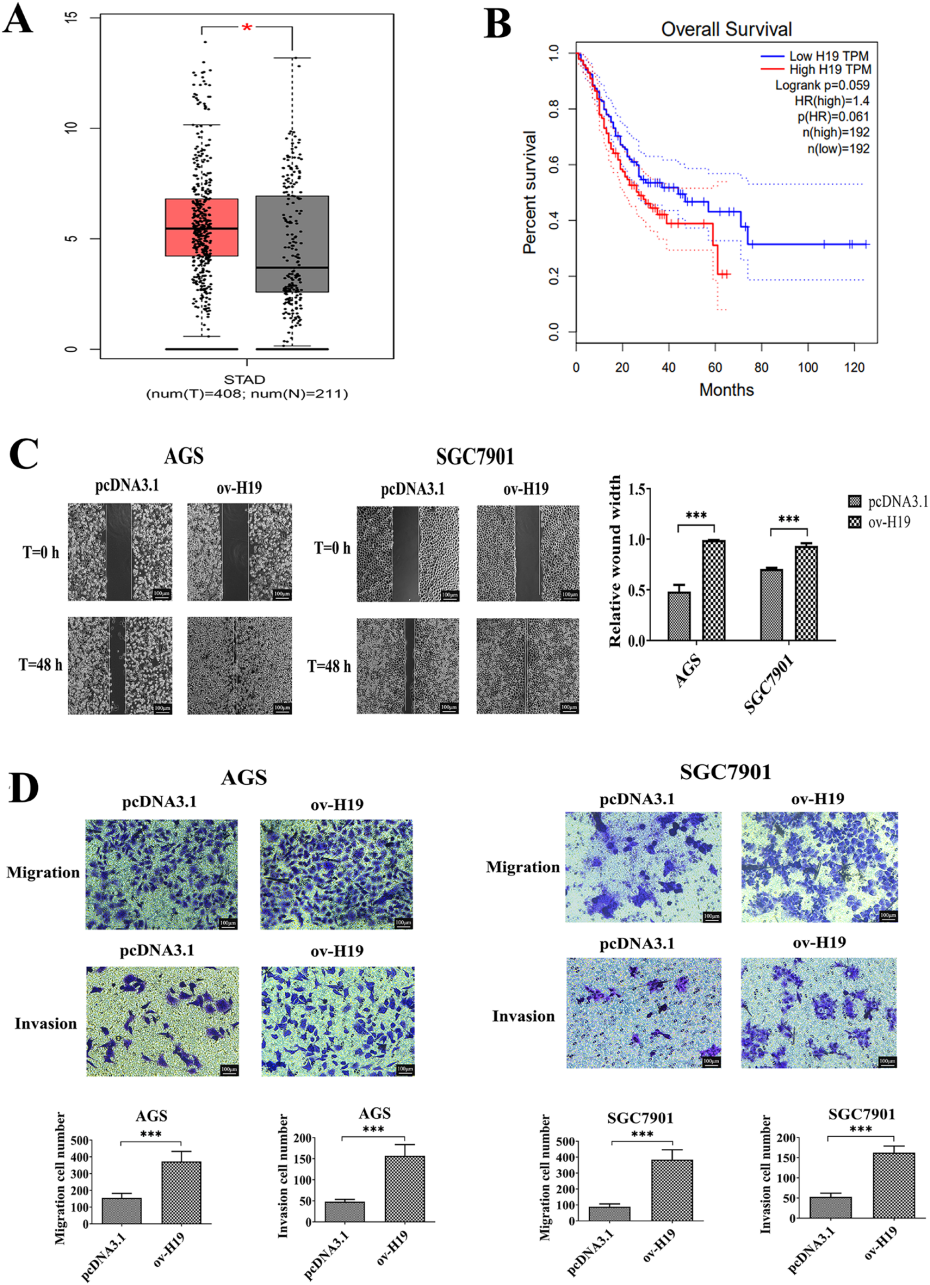
The expression of H19 in GC tissues, its correlation with the prognosis of patients with GC, and its impact on cell invasive and migration. (**A**) Boxplot showing H19 expression in 408 GC tissues and 211 normal gastric tissues [data from The Cancer Genome Atlas and GTEx]. (**B**) Kaplan–Meier analysis of relationship between H19 expression and prognosis of gastric cancer. *p < 0.05. AGS and SGC7901 cells were transfected with pcDNA3.1-H19 for 48 h. (**C**) Wound healing assay was performed to verify GC cell migration; (**D**) transwell assay was conducted to examine GC cell migration and invasion. Each experiment was carried out in triplicate. pcDNA3.1, pcDNA3.1 empty vector transfected cells, ov-H19, pcDNA3.1-H19 transfected cells. *p < 0.05; **p < 0.01; ***p < 0.001.

To identify H19s potentially involved in gastric cancer progression, we analysed H19 expression profiles using the GEPIA II databases. The results showed that H19 was overexpressed in gastric cancer tissues compared to adjacent normal tissues (Fig. 4A). We further investigated whether H19 expression correlated with the outcome of gastric cancer patients. Kaplan–Meier survival estimates showed that high H19 expression in gastric cancer tissues was significantly associated with worse overall survival (p = 0.061, log-rank test) (Fig. 4B). Analysis of H19 expression in gastric cancer patients with different clinicopathological characteristics showed that high or low H19 expression was positively correlated with pathological parameters such as T stage and AJCC stage (all p < 0.05, Table 2). The study also investigated the effects of H19 on invasion, migration and proliferation of GC cells. The results of Transwell and wound healing assays showed that the cells transfected with ov-H19 (pcDNA3.1-H19) exhibited a significant increase in wound healing, migration and invasion activity compared to the cells transfected with empty vector (pcDNA3.1). (Fig. 4C,D). These results indicated that increased expression of H19 may play a pivotal role in the pathogenesis of gastric cancer and the malignant outcome of patients with gastric cancer.

**Table 2:** The relationship between the expression level of H19 and the clinicopathological parameters of GC.

Clinicopathological feature	H19		c2	*p*
	Low (n = 207)	High (n = 208)		
Gender				
Male	136	131	0.3345	0.563
Female	71	77		
Age (t/a)				
> 60	138	140	0.212	0.8994
≤ 60	66	66		
Unknown	3	2		
T stage				
T1 + T2	73	40	16.55	0.0003*
T3 + T4	128	166		
TX	6	2		
N stage				
N0	63	62	2.57	0.766
N1	51	59		
N2	42	37		
N3	39	43		
NX	11	6		
Unknown	1	1		
M stage				
M0	184	183	0.08497	0.9584
M1	13	14		
Mx	10	11		
AJCC stage				
I	39	20	14.04	0.0072*
II	51	71		
III	81	89		
IV	19	21		
Unknown	17	7		

(*p < 0.05, **p < 0.01), TNM according to staging TNM of American Joint Committee on Cancer (AJCC) in 2010.

### H19 knockdown reduces *H. pylori*-induced cell proliferation, migration, and invasion

**Figure 5:**
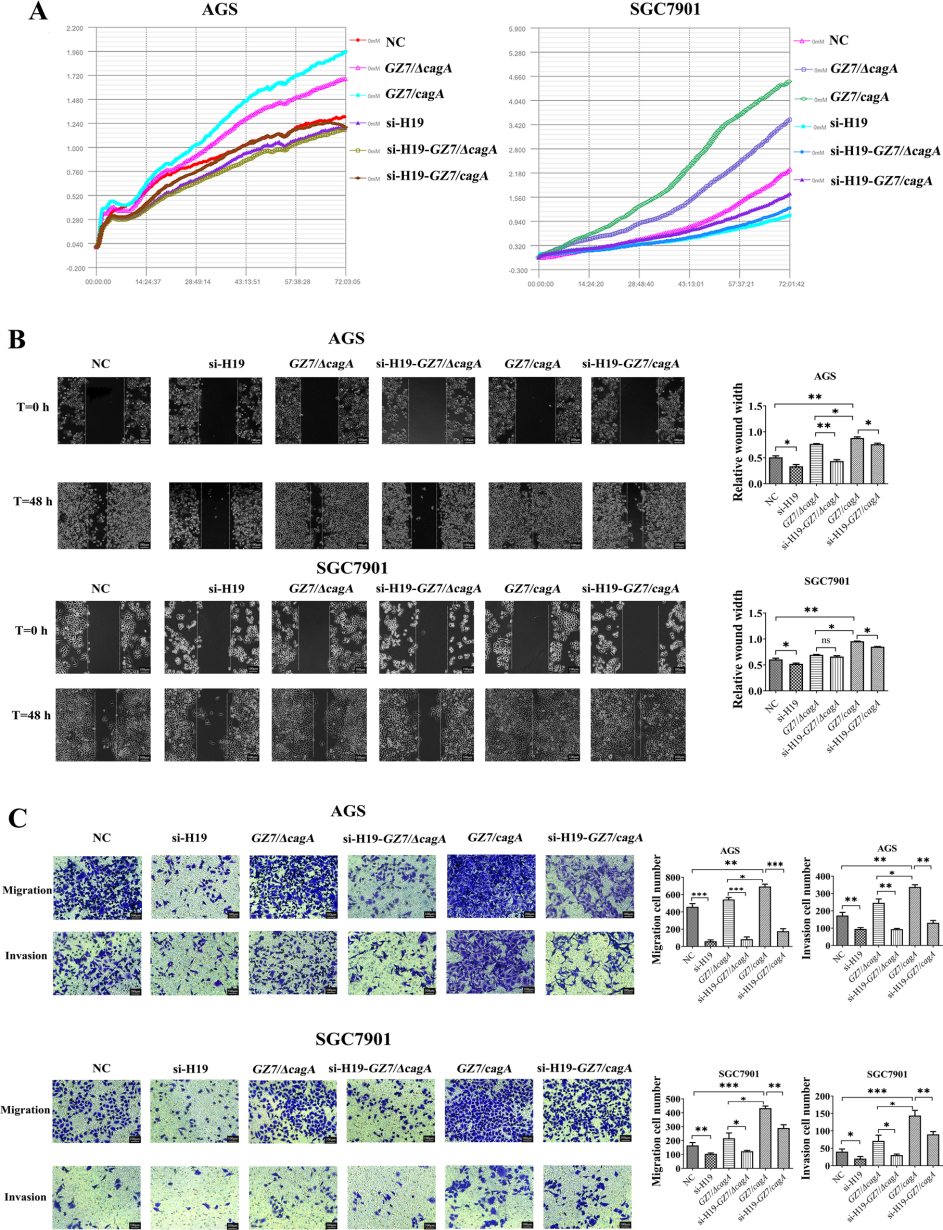
H19 knockdown inhibited invasion and migration induced by *H. pylori* CagA. After transfecting AGS and SGC7901 cells with si-H19 for 24 h, GZ7/ΔcagA and GZ7/cagA strains were infected for 24 h at an MOI of 60. (**A**) RTCA assay was performed to verify GC cell proliferation; (**B**) Wound healing assay was conducted to examine GC cell migration; (**C**) transwell assay was carried out to analyze GC cell migration and invasion. *NC* si-H19 control transfected cells, *si-H19* si-H19 transfected cells, *GZ7/ΔcagA* GZ7/ΔcagA strains infected cells, *GZ7/cagA* GZ7/cagA strains infected cells, *si-H19-GZ7/ΔcagA* GZ7/ΔcagA strains infect si-H19-transfected cells, *si-H19-GZ7/cagA* GZ7/cagA strains infect si-H19-transfected cells. *p < 0.05; **p < 0.01; ***p < 0.001.

To gain a more complete understanding of the cellular behaviour of *H. pylori*-infected cells, transfection of AGS and SGC-7901 cells with si-H19 was performed prior to infection with GZ7/ΔcagA and GZ7/cagA strains. RTCA assay of cell proliferation showed that si-H19 transfection significantly decreased the proliferation ability of cells compared to the normal control group, and H19 knockdown significantly inhibited cell proliferation induced by GZ7/ΔcagA and GZ7/cagA infection (Fig. 5A). Furthermore, H19 knockdown also reduced the healing ability of GZ7/ΔcagA and GZ7/cagA infected cells based on the results of the wound healing assay (Fig. 5B). Transwell assay showed that H19 knockdown significantly decreased the invasion and migration ability of GZ7/ΔcagA and GZ7/cagA infected cells (Fig. 5C). Furthermore, GZ7/cagA-infected cells showed more pronounced effects compared to GZ7/ΔcagA-infected cells. These results demonstrated that H19 knockdown reduced *H. pylori*-induced cell proliferation, migration and invasion, indicating its potential involvement in these processes.

### H19-interacting protein screening and identification

The present results suggest that H19 may play a critical role in *H. pylori*-associated GC. To investigate the possible interaction of H19 with specific proteins, biotin-labelled antisense-H19 magnetic beads and biotin-labelled sense-H19 magnetic beads were used. The beads were used in RNA pull-down assays in conjunction with proteins from AGS cells infected with GZ7/cagA and uninfected cells. The aim was to investigate whether H19 could function by interacting with specific proteins. After sending the magnetic beads to Shanghai Biotech for mass spectrometry analysis, a total of 20 differentially expressed proteins bound to H19 were identified in the GZ7/cagA-infected group (sense-H19-*H. pylori*) compared to the uninfected group (sense-H19; Table 3). A literature search of 20 H19 target proteins revealed that the YWHAZ protein is linked with cell cycle control and tumorigenesis. YWHAZ, also known as 14-3-3ζ, encodes an adapter protein from the 14-3-3 family. It affects a number of signalling pathways, including cell proliferation, cell cycle, apoptosis, migration, and invasion, and is critical in tumorigenesis^26,27^. Therefore, we postulate that *H. pylori* may be implicated in carcinogenesis by suppressing YWHAZ expression through H19.

**Table 3:** Differentially expressed proteins of sense-H19-*H. pylori*/sense-H19.

Gene	Mw(kD)	log2(sense-H19-*H. pylori*/sense-H19)	log2(Mean SP)	Diff sig
PRSS3P2	26.537	5.1293	4.1699	++
ANXA2	38.604	− 3.1699	2.3219	−
ANXA2P2	38.659	2.8074	2	++
MRPL12	21.348	2.585	1.8074	++
DNAJC9	29.91	2.585	1.8074	++
HNRNPA3	39.595	− 2.3219	1.585	−
DSC1	99.987	− 1.3219	1.8074	−
SSB	46.837	2	1.3219	+
QARS	87.799	− 2	1.3219	−
SUB1	14.395	− 2	1.3219	−
MYL6	16.93	2	2.3219	++
YWHAZ	27.745	− 2	1.3219	−
SRSF4	56.678	2	1.3219	+
FAM98B	45.547	2	1.3219	+
CALML5	15.893	1.9069	3.2479	++
RPL12	17.819	− 1.585	2	−
MYH9	226.532	1.3219	1.8074	+
FABP5	15.164	1.3219	3.8074	+
EIF2S1	36.112	− 1.1699	3.7004	−
SERBP1	44.965	1.1699	2.7004	+

Compared with the control group, “+” marks the up-regulated proteins; “−” marks the down-regulated proteins.

### H19 can affect the distribution of cell cycle upon* H. pylori *infection via YWHAZ

**Figure 6:**
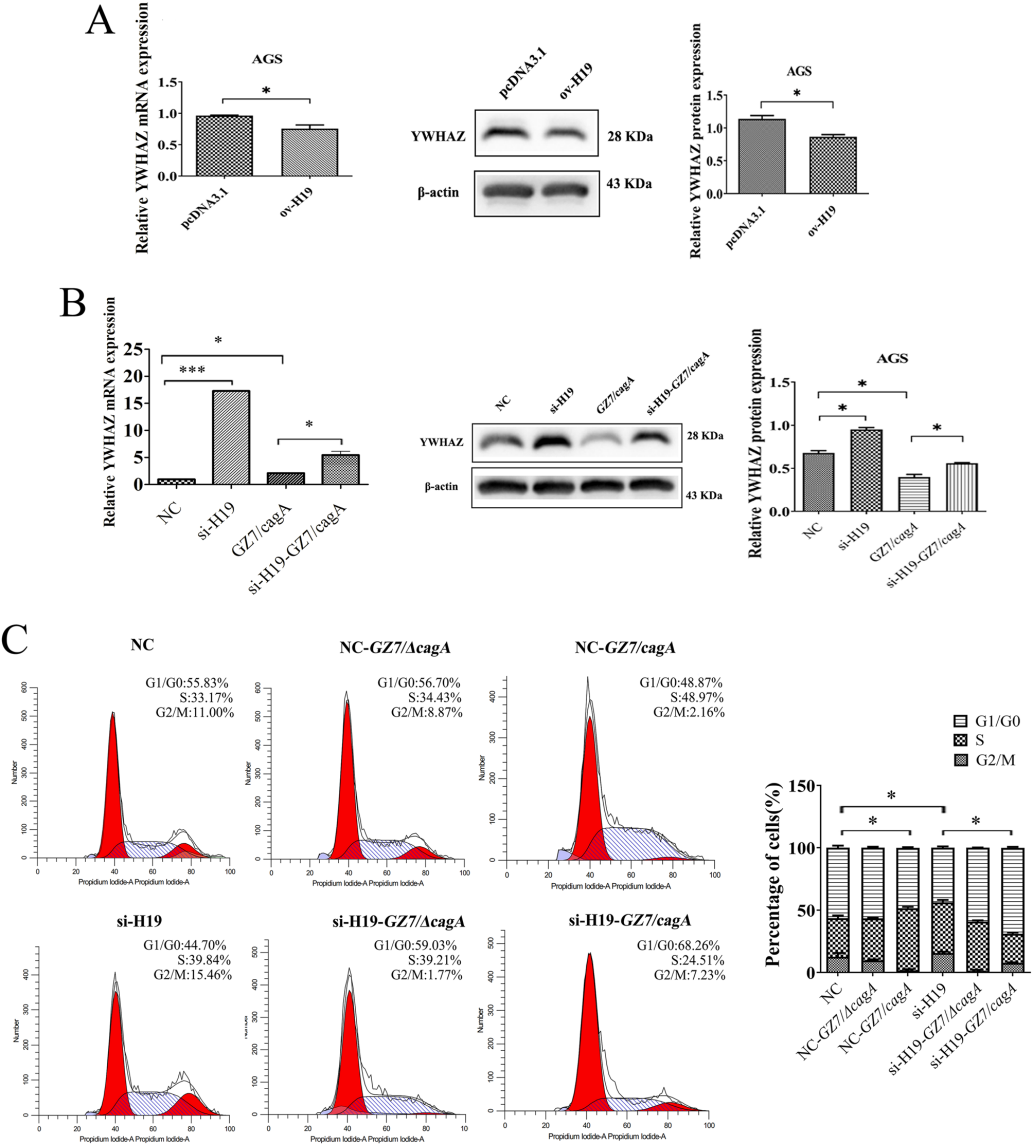
Effects of *H. pylori* and H19 on YWHAZ expression and cell cycle distribution. (**A**) YWHAZ mRNA and protein expression in pcDNA3.1-H19 transfected cells; (**B**) mRNA and protein expression of YWHAZ in si-H19 transfected and *H. pylori*-infected cells. (**C**) Effect of si-H19 transfected on the cell cycle distribution of *H. pylori*-infected cells. *pcDNA3.1* pcDNA3.1 empty vector transfected cells, *ov-H19* pcDNA3.1-H19 transfected cells, *NC* si-H19 control transfected cells, *si-H19* si-H19 transfected cells, *GZ7/ΔcagA* GZ7/ΔcagA strains infected cells, *GZ7/cagA* GZ7/cagA strains infected cells, *si-H19-GZ7/ΔcagA* GZ7/ΔcagA strains infect si-H19-transfected cells, *si-H19-GZ7/cagA* GZ7/cagA strains infect si-H19-transfected cells *p < 0.05; **p < 0.01; ***p < 0.001.

To investigate the effect of *H. pylori* and H19 on YWHAZ expression, pcDNA3.1-H19 was introduced into AGS cells and allowed to grow for 48 h. It was observed that overexpression of H19 could lead to the downregulation of YWHAZ, as confirmed by RT-qPCR and Western blotting analysis (Fig. 6A). Furthermore, AGS cells were transfected with si-H19 and subsequently infected with GZ7/cagA (MOI 60:1) for 24 h to analyse YWHAZ expression. The results showed that YWHAZ expression was higher in cells transfected with si-H19. However, infection with GZ7/cagA decreased the expression of YWHAZ. Interestingly, GZ7/cagA infection counteracted the upregulation of YWHAZ expression induced by si-H19. These findings imply that YWHAZ, as an H19 binding protein, may play an important role in the pathogenic process associated with *H. pylori* infection (Fig. 6B). Since YWHAZ is closely linked to the cell cycle, the effect of H19 knockdown on the cell cycle distribution of *H. pylori*-infected cells was investigated. The results showed that transfection of si-H19 into the cells increased the number of cells entering the S phase and G2 compared to the control group. In addition, *H. pylori* infection of si-H19-transfected cells increased their entry into the G2 phase, suggesting that knockdown of H19 improved the cell cycle block caused by *H. pylori* infection. These results further confirmed that H19 could regulate cell cycle distribution by *H. pylori* by using its target protein YWHAZ (Fig. 6C).

## Discussion

GC is one of the leading causes of cancer death worldwide and is associated with high morbidity. Numerous studies have shown that GC is strongly associated with atrophic gastritis due to H. pylori infection^28^. *H. pylori* secretes numerous virulence factors that have the potential to disrupt intracellular signalling pathways in the host, thereby facilitating the onset of neoplastic transformation. Among these factors, cytotoxin-associated gene A (CagA), vacuolating cytotoxin A (VacA) and outer membrane proteins (OMPs) have been implicated in the development of gastric cancer^29^. All *H. pylori* isolates secrete vacuolating cytotoxin A (VacA) via the type V secretion system. VacA has several effects on the host cell, including cell vacuolization, alteration of mitochondrial membrane permeability, inhibition of T-lymphocyte activation and proliferation, and activation of cell signalling^30^. *H. pylori* attach to the gastric epithelial cell and transfer the CagA protein into the cell via the OMPs, which is associated with gastric cancer^2^. CagA-induced cellular changes relevant to cancer pathogenesis include inhibition of apoptosis, stimulation of cell proliferation, degradation of the p53 tumour suppressor and double-strand DNA breaks. It is thought that cagA-positive strains are associated with more severe inflammation, higher levels of atrophy and a greater likelihood of progression to gastric adenocarcinoma than cagA-negative strains^31^. To date, there has been relatively little progress in determining the pathogenesis of GC by *H. pylori* CagA^32^.

H19 is widely expressed in GC tissues and plays an important role in the progression of GC through multiple pathways^33^. The results of the present study showed a significant increase in H19 expression and cytoplasmic localisation in *H. pylori* infected cells, consistent with the observations of Zhang et al.^10^. Another notable finding was that the GZ7/cagA strain showed a pronounced upregulation of H19 expression compared to the GZ7/∆cagA strain. This suggests a possible link between the upregulation of H19 and the pathogenicity factor CagA in *H. pylori* infection. Research has shown that the localisation of lncRNAs within cells can significantly affect the stability and translation of mRNA in the cytoplasm, allowing them to perform specific functions^34^. According to a study by Yang et al.^35^, H19 was found to be predominantly located in the cytoplasm of keratin-forming cells and may exert pro-inflammatory effects by activating the NF-κB signalling pathway. Therefore, it was postulated that H19 overexpression induced by *H. pylori* infection may also act predominantly in the cytoplasm and be involved in its pathogenic process.

DDR involves activating ATM and ATR, phosphorylating H2AX histones at Ser 139 (γ-H2AX), activating sensors Chk1 and Chk2, and loading the HR core repair protein RAD51, which activates p53^36^. p53 plays a central role in responding to DNA damage and determines the outcome of the DNA damage checkpoint response by regulating cell cycle arrest and apoptosis^37^. Two main mechanisms are used for DSB repair: non-homologous end joining (NHEJ) and homologous recombination (HR). In the HR pathway, RAD51 catalyses this process by promoting strand-invasion of a resected 3ʹ single-stranded DNA (ssDNA) end into the homologous repair template; mutational inactivation or abnormal RAD51 production can cause genomic phenotypic instability, which in turn increases the risk of spontaneous DNA damage and cancer^38^. H19 has previously been proposed to regulate the DNA damage response; mass spectrometry analysis of potential H19-interacting proteins revealed several DNA damage response and repair molecules directly associated with H19, but the underlying mechanisms remain to be defined^39,40^. The current study found that H19 overexpression significantly increased the expression of γH2A.X and p-ATM, two essential proteins involved in DNA damage, and also reduces the expression of Rad51 and p53, two proteins involved in DNA repair. These observations suggest that H19 overexpression could potentially increase DNA damage while limiting repair capacity, which is consistent with the findings of Wang et al.^41^, who identified the ability of H19 to cause DNA damage in hepatocellular carcinoma cells. *H. pylori* infection is associated with increased DNA damage. CagA may act as a critical regulator of DNA damage repair in *H. pylori* infection, altering the balance between DNA damage and repair, leading to genomic instability and cancer development^42,43^. In view of this, RNA interference methods were used to generate AGS and SGC7901 cell models with reduced levels of H19 expression, which were subsequently infected with GZ7/ΔcagA and GZ7/cagA strains, respectively, to detect the expression of γH2A.X, p-ATM, P53, and Rad51. The results of Western blotting and immunofluorescence assays suggested that knockdown of H19 expression could effectively inhibit the *H. pylori* CagA-induced DNA damage response in GC cells. However, the effect of CagA-regulated H19 expression on DNA damage repair might not be related to the Rad51 repair mechanism. The results suggest that the increased expression of H19 driven by *H. pylori* CagA may play an important role in the DNA damage response induced by *H. pylori*.

In the current study, it was observed that H19 overexpression could significantly enhance the invasion and migration of GC cells, which was consistent with the previous finding that H19 could promote invasion and migration via involvement in the miR-138/E2F2 axis in GC, as proposed by Yu et al.^44^. Further investigation showed that H19 knockdown in GC cells reduced proliferation, invasion, and migration capabilities. Interestingly, H19 knockdown effectively reduced cell proliferation, invasion, and migration generated by GZ7/ΔcagA and GZ7/cagA infection. Notably, the effect was more pronounced in the GZ7/cagA-infected group than in the GZ7/∆cagA-infected group. The results suggest that *H. pylori* CagA infection can modulate GC cell proliferation, invasion and migration by altering H19 expression.

It is importance to identify the specific proteins that interact with the lncRNA H19. To achieve this RNA pull-down, combined mass spectrometry was used to identify H19-binding proteins associated with *H. pylori* infection. This can facilitate the study of individual proteins that interact with H19 and may play a role in the development of GC associated with *H. pylori* infection. A total of 20 differentially expressed H19-binding proteins were identified in the GZ7/cagA infected and GZ7/cagA uninfected groups. One of these proteins, YWHAZ, has been reported to be closely associated with various signalling transduction pathways and the cell cycle. It plays a critical role in DNA damage repair and carcinogenesis^45,46^. A more detailed analysis of the effects of *H. pylori* infection and H19 on YWHAZ expression was performed. The results showed that GZ7/cagA infection decreased YWHAZ expression, which is consistent with the observed reduction in YWHAZ expression in cells overexpressing H19. In contrast, YWHAZ expression was upregulated in H19 knockdown cells, and GZ7/cagA infection could respond to the high YWHAZ expression caused by H19 knockdown. This suggests that YWHAZ may play a role in the development of H. pylori-related pathogenesis as a protein that binds to H19.

*H. pylori* infection can induce genetic instability by damaging DNA, leading to cell cycle arrest and apoptosis in gastric epithelial cells. The results of further experiments confirmed that cells infected with GZ7/cagA were significantly blocked in the S phase. These findings were consistent with the results of Gao’s^47^ on the induction of G1/S arrest in AGS cells upon *H. pylori* infection. H19 knockdown cells showed an increase in S phase and G2, consistent with the observations made by Ping Liu et al.^48^ in their study on glioma cells after knockdown of H19. Further analysis showed that H19 knockdown alleviated GZ7/cagA infection-induced S phase arrest and facilitated cell entry into the G2 phase, thereby improving cell cycle progression. However, further experiments are required to confirm the role of YWHAZ in this process.

## Conclusions

In this current study, *H. pylori* infection can lead to the upregulation of H19 gene expression, which subsequently affects the DNA damage response and facilitates the invasion and migration of GC cells. However, the knockdown of H19 can significantly inhibit the DNA damage response and the migratory and invasive ability of GC cells induced by *H. pylori* infection. In addition, H19 was found to be involved in cell cycle arrest induced by *H. pylori* infection through the regulation of its binding protein YWHAZ. The CagA+ strains induced a stronger biological response in this process than the CagA knockdown strains. Thus, by regulating the expression of H19, *H. pylori* CagA can effectively modulate the DNA damage repair response and promote proliferation, migration, and invasion of GC cells.

### Supplementary Information

Supplementary Information.

## Data Availability

The dataset supporting the conclusions of this article is included within the article.
