# Supplementary material for: A prominent role of LncRNA H19 in H. pylori CagA induced DNA damage response and cell malignancy
Source: Sci Rep. 2024 Jun 20;14:14185. doi: 10.1038/s41598-024-65221-y (PMC11190245; doi:10.1038/s41598-024-65221-y)
Supplement: Supplementary file 1 — Supplementary Information. [file 41598_2024_65221_MOESM1_ESM.pdf]

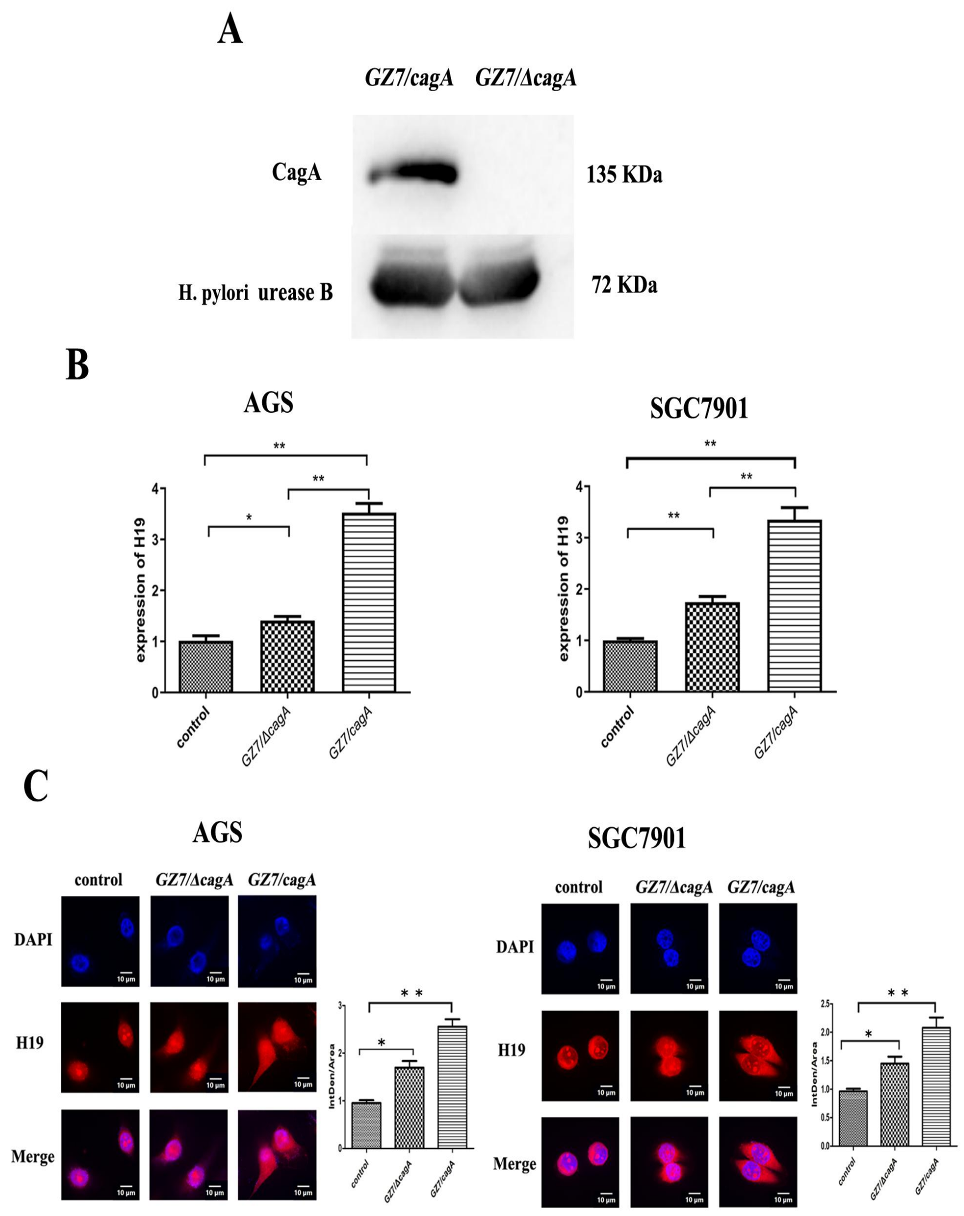

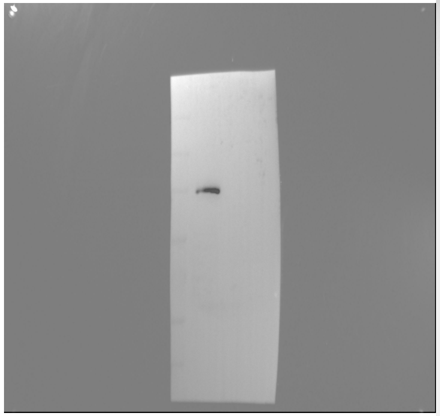

Fig 1 A-cagA

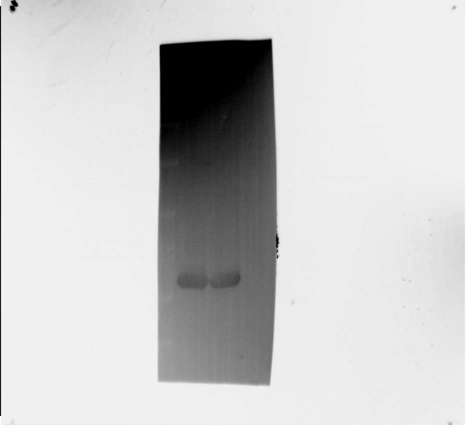

Fig 1 A-Hp,Urease B

Figure.1

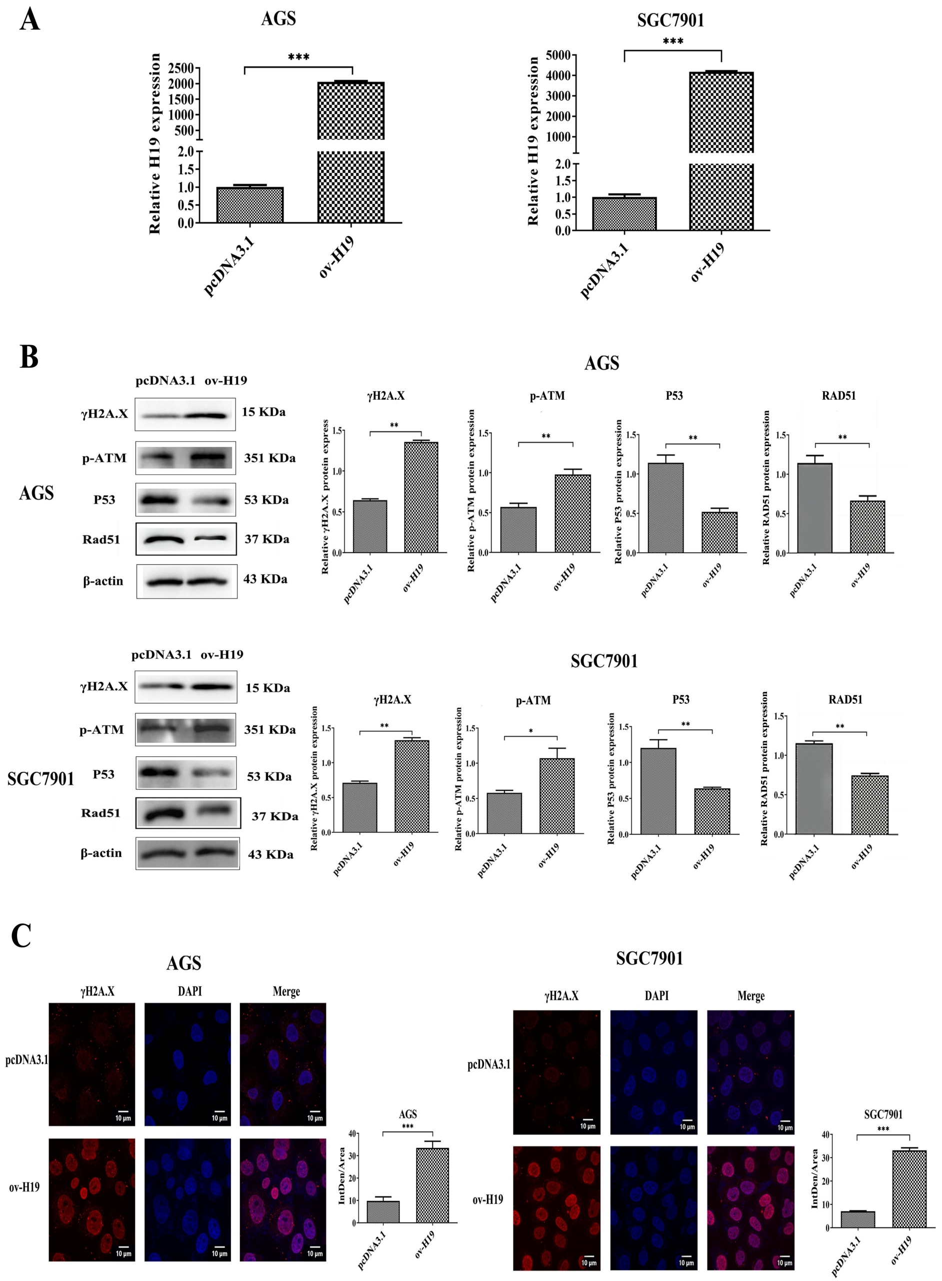

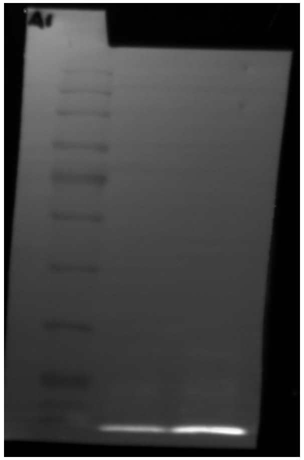

Fig 2B-AGS-rH2AX

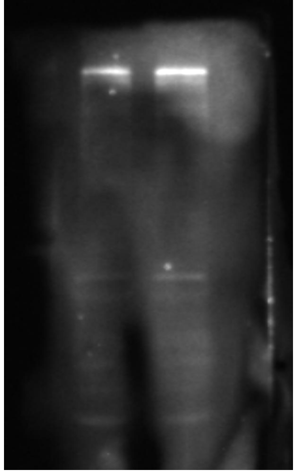

Fig 2B-AGS-p-ATM

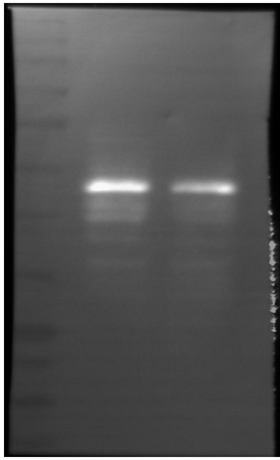

Fig 2B-AGS-P53

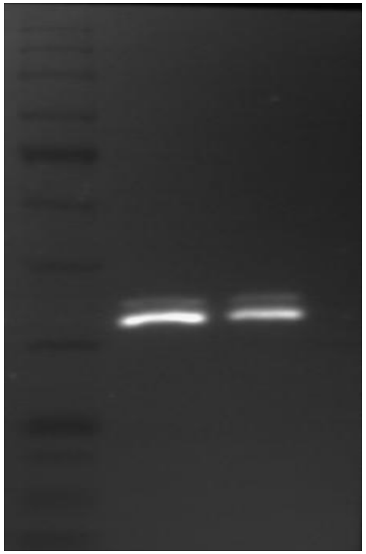

Fig 2B-AGS-RAD51

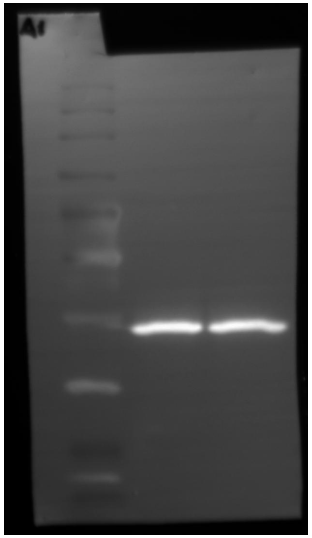

Fig 2B-AGS-actin

AGS

Figure.2

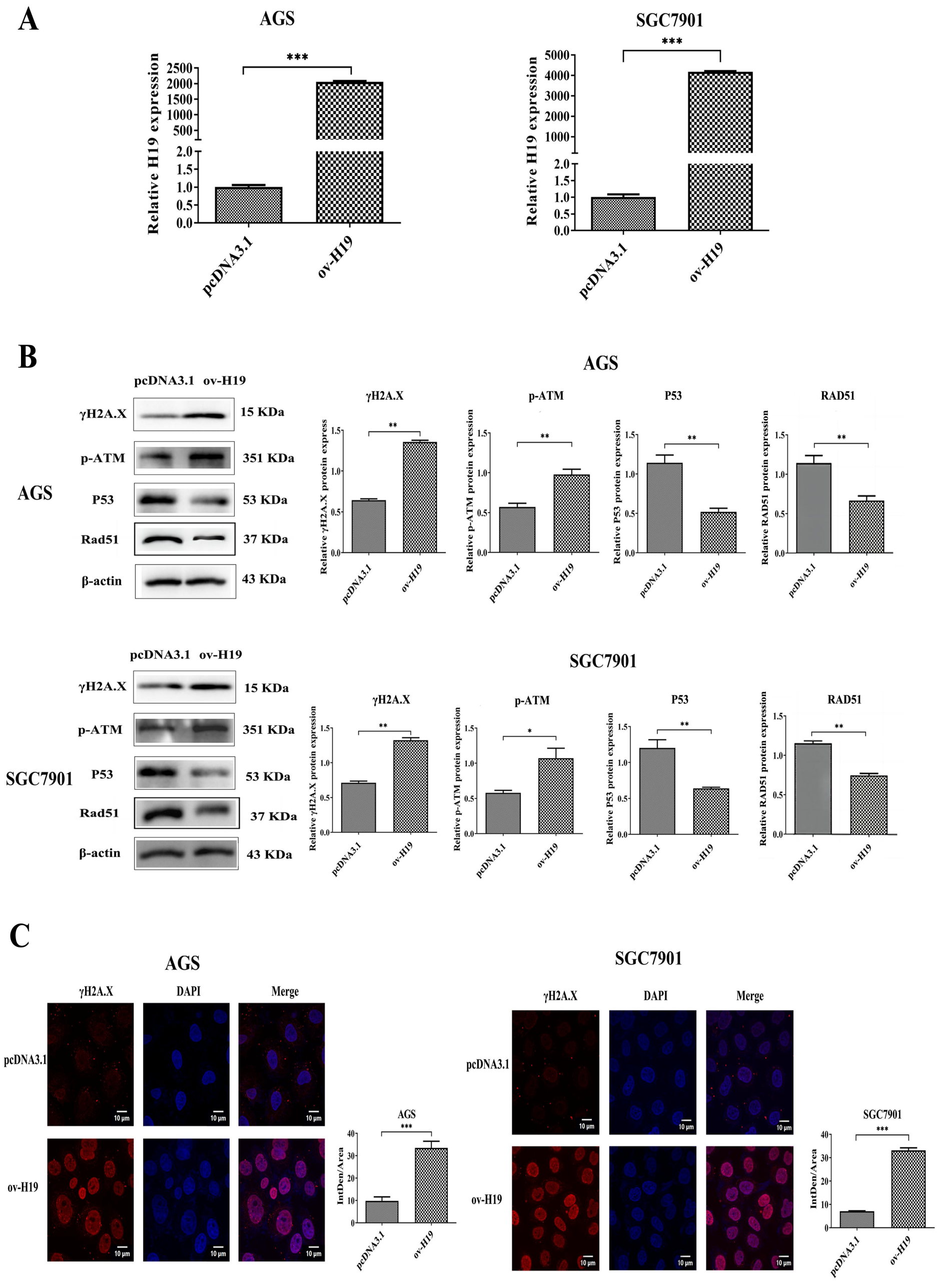

Figure.2

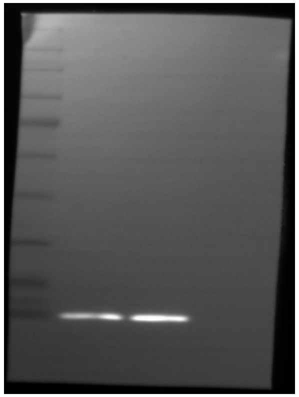

Fig 2B-SGC-7901-rH2AX

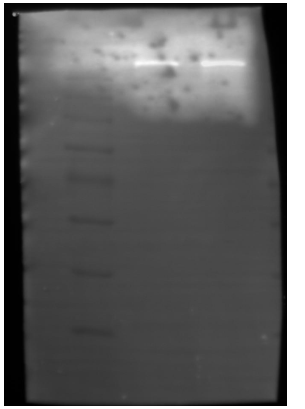

Fig 2B-SGC-7901-p-ATM

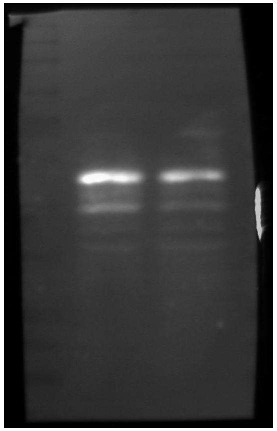

Fig 2B-SGC-7901-P53

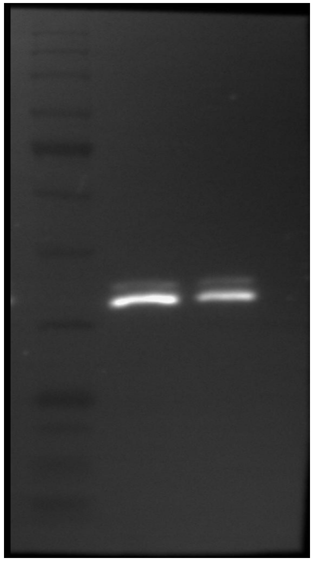

Fig 2B-SGC-7901-RAD51

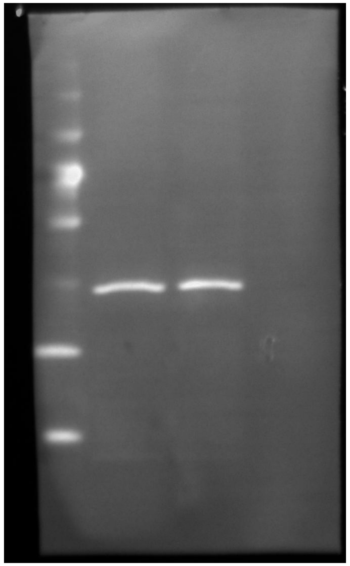

Fig 2B-SGC-7901-actin

SGC-7901

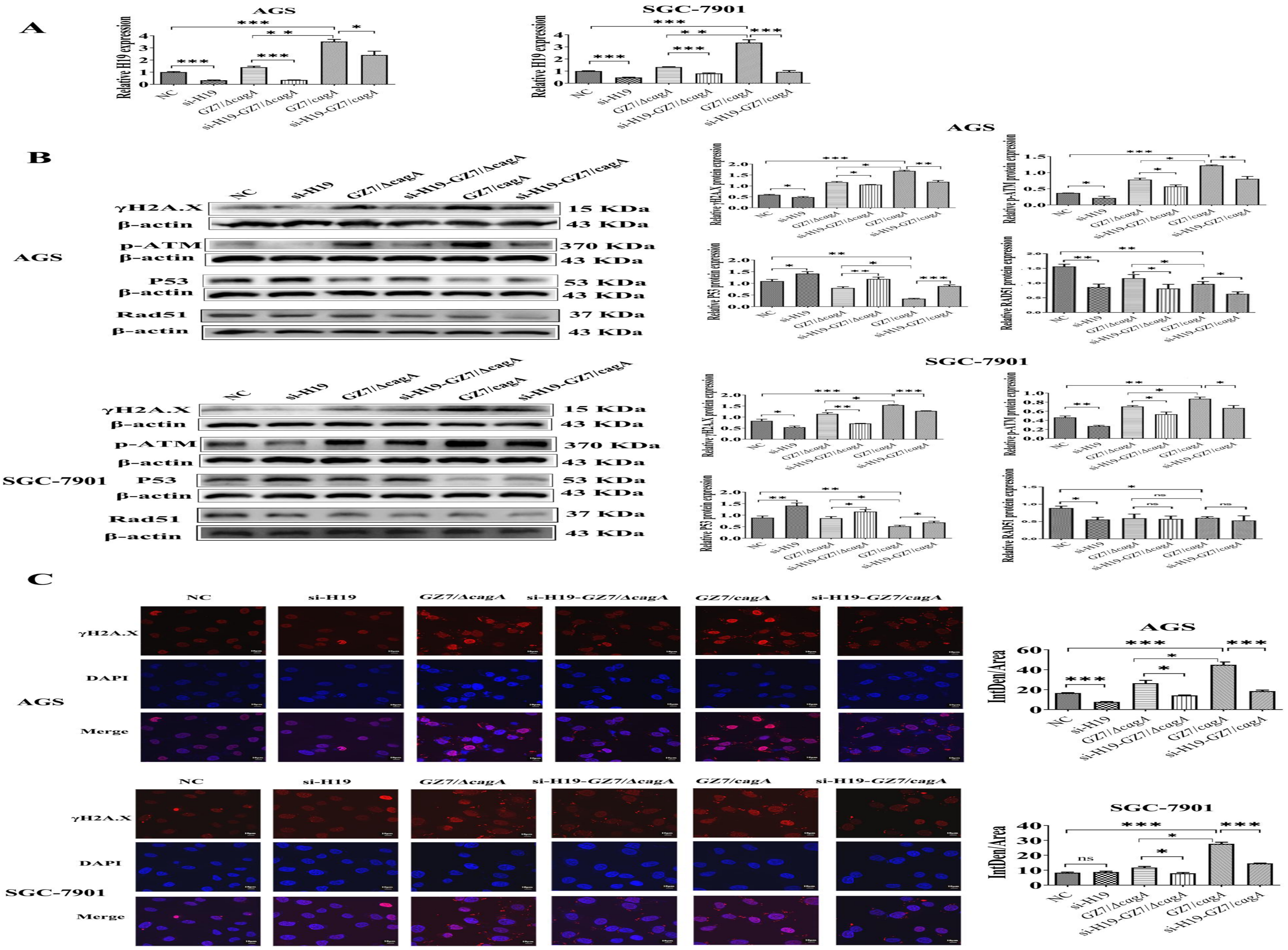
A
AGS
SGC-7901
Relative H19 expression
NC
si-H19
GZ7/ΔcagA
si-H19-GZ7/ΔcagA
GZ7/cagA
si-H19-GZ7/cagA
B
γH2A.X
β-actin
p-ATM
P53
Rad51
15 KDa
43 KDa
370 KDa
53 KDa
37 KDa
C
DAPI
Merge
IntDen/Area

Figure.3

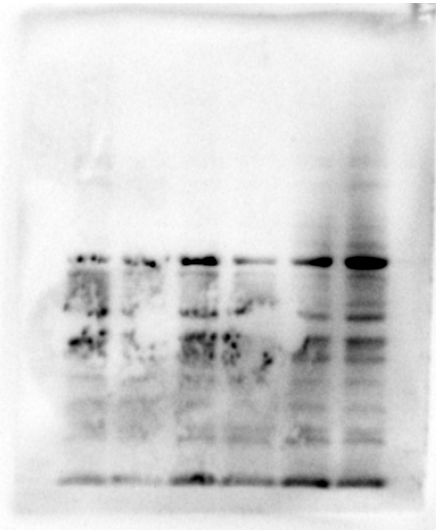

Fig 3B-AGS-rH2AX

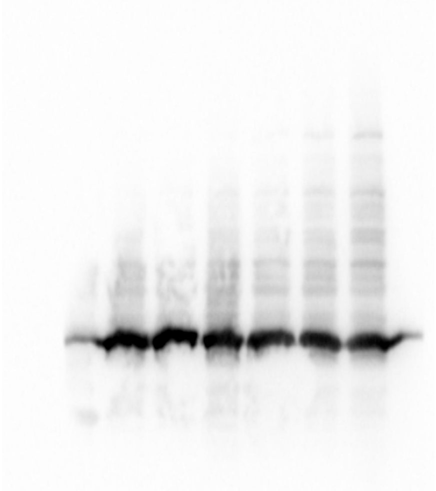

Fig 3B-AGS-rH2AX-actin

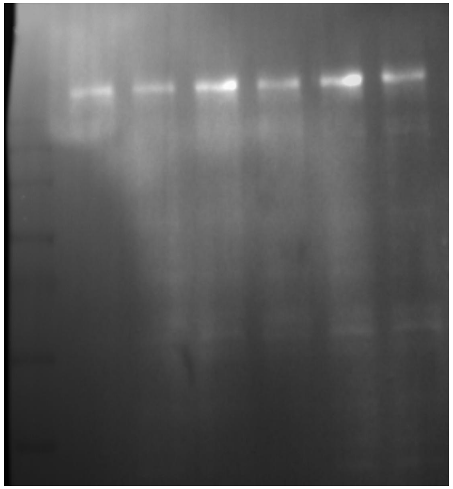

Fig 3B-AGS-p-ATM

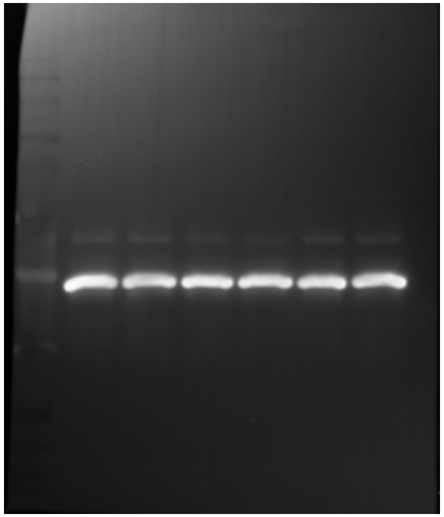

Fig 3B-AGS-p-ATM-actin

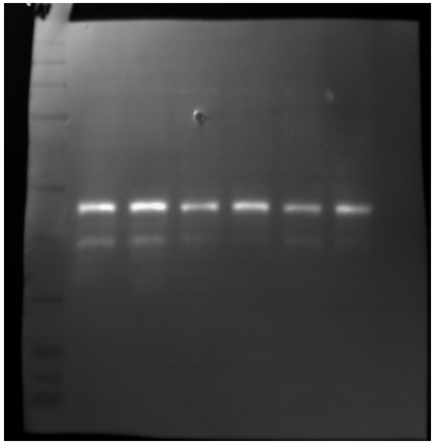

Fig 3B-AGS-p53

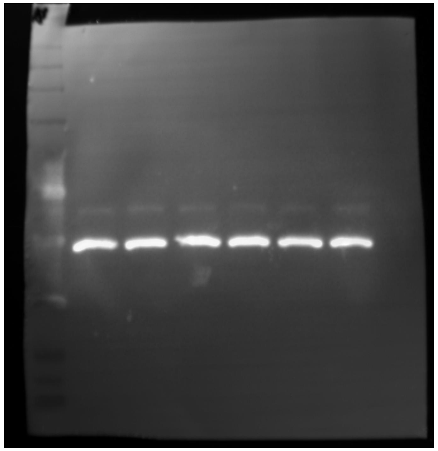

Fig 3B-AGS-p53-actin

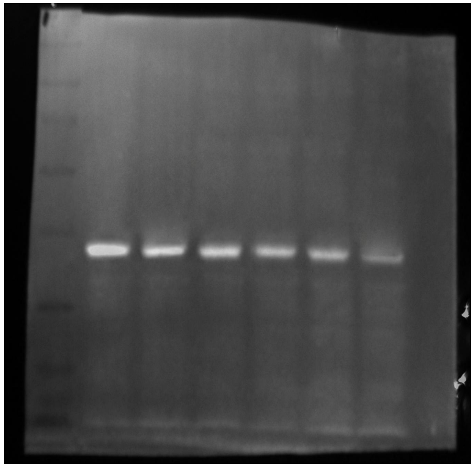

Fig 3B-AGS-RAD51

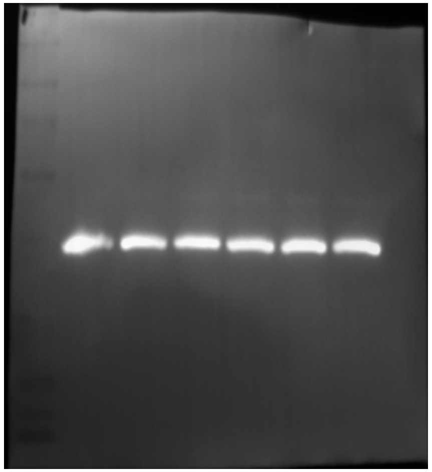

Fig 3B-AGS-RAD51-actin

Fig 3B-AGS

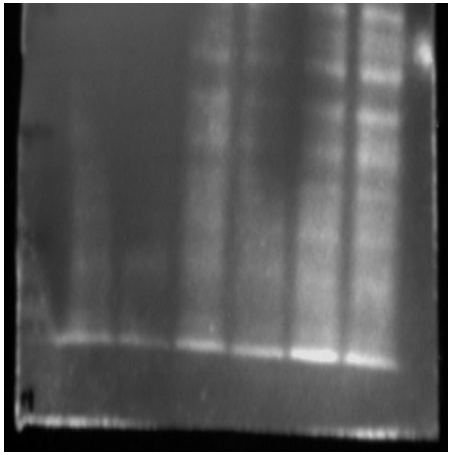

Fig 3B-SGC7901-rH2AX

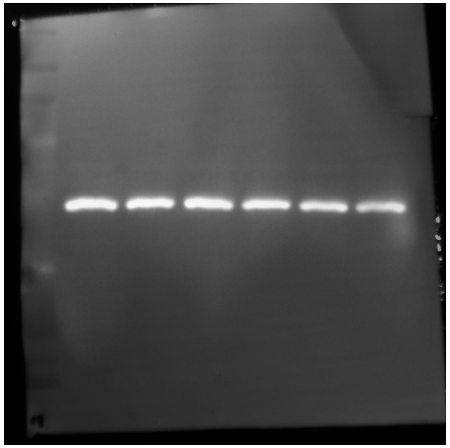

Fig 3B-SGC7901-rH2AX-actin

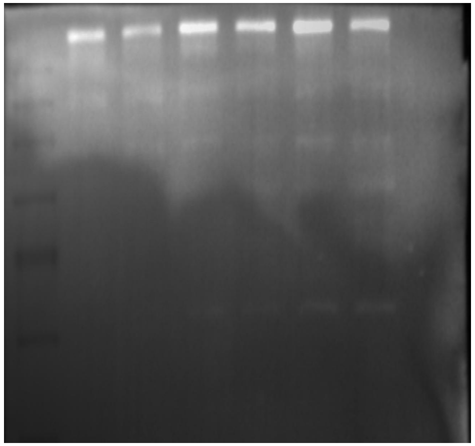

Fig 3B-SGC7901-p-ATM

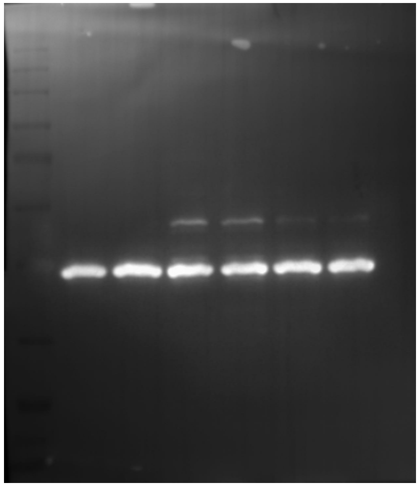

Fig 3B-SGC7901-p-ATM-actin

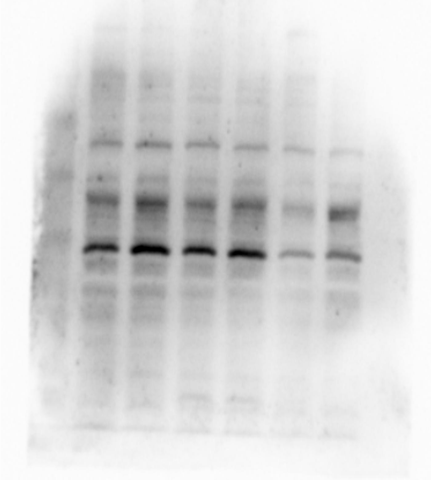

Fig 3B-SGC7901-p53

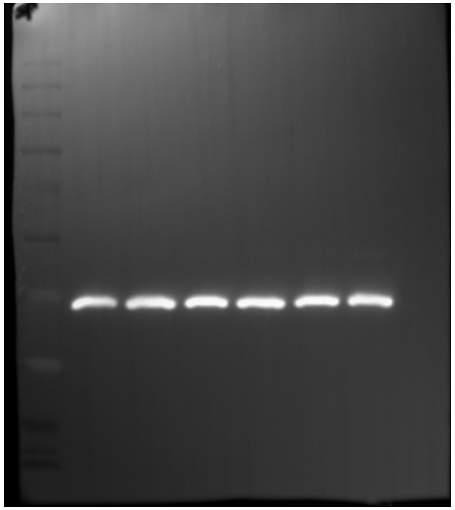

Fig 3B-SGC7901-p53-actin

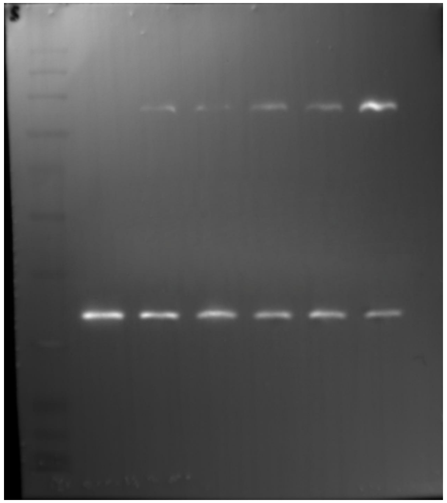

Fig 3B-SGC7901-RAD51

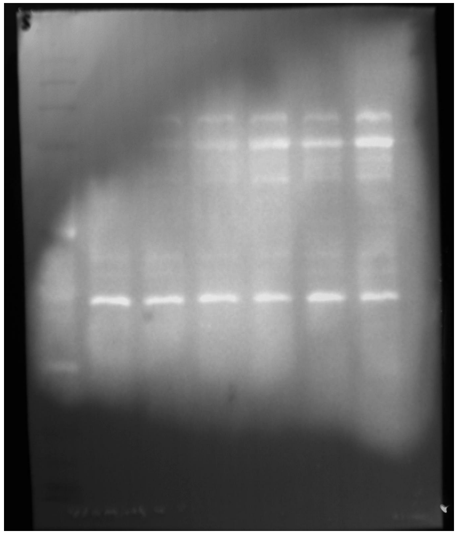

Fig 3B-SGC7901-RAD51-actin

Fig 3B-SGC-7901

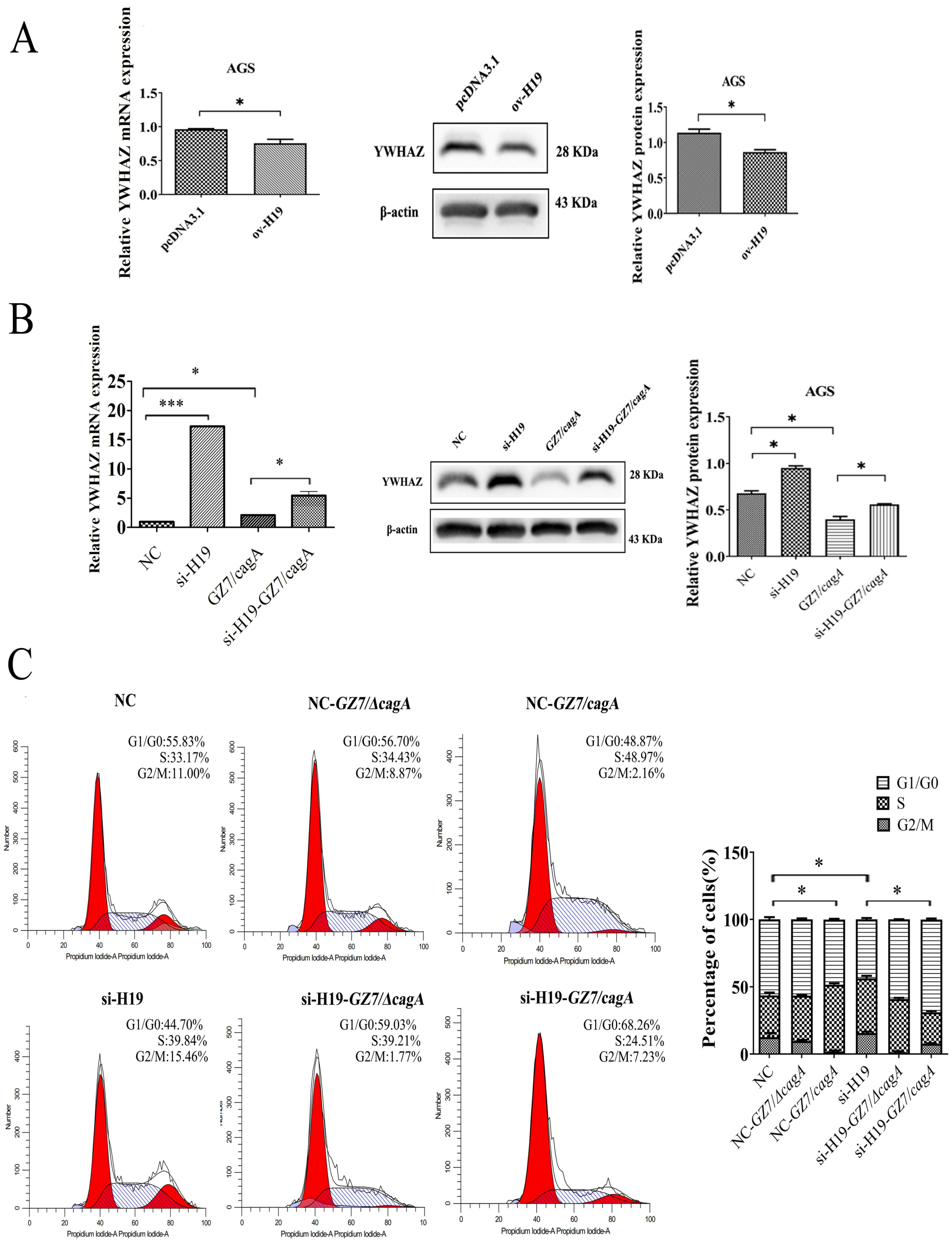

Figure.6

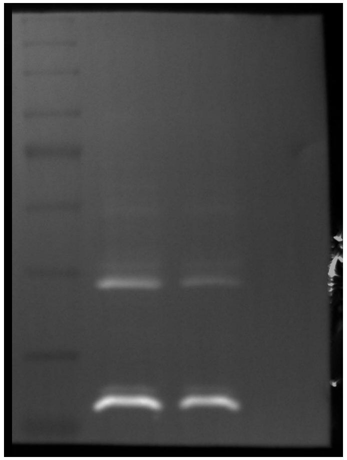

Fig 6A-AGS-YWHAZ

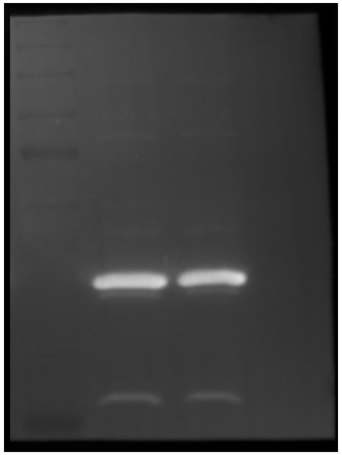

Fig 6A-AGS-YWHAZ-actin

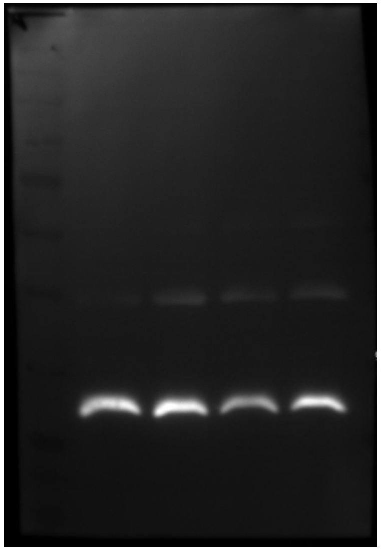

Fig 6B-AGS-YWHAZ

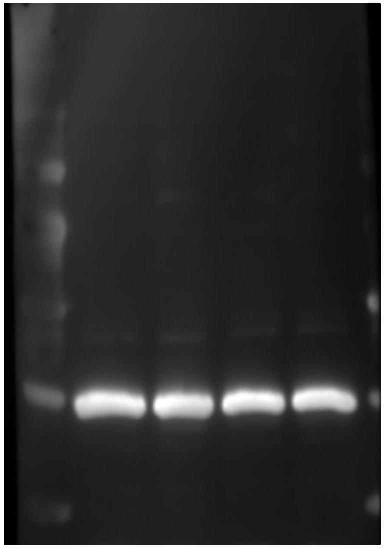

Fig 6B-AGS-YWHAZ-actin
